# Molecular Characterization and Pathogenicity of *Colletotrichum falcatum* Causing Red Rot on Sugarcane in Southern Florida

**DOI:** 10.3390/jof10110742

**Published:** 2024-10-27

**Authors:** Fernanda Rodrigues Silva, Mário Lúcio V. de Resende, Larissa Carvalho Ferreira, Olamide Adesina, Katia V. Xavier

**Affiliations:** 1 Department of Plant Pathology, University of Florida, Everglades Research and Education Center, Belle Glade, FL 33430-4702, USA; frodriguezsilva@ufl.edu (F.R.S.); larissacarvalhof@ufl.edu (L.C.F.); oadesina@ksu.edu (O.A.); 2 Departamento de Química/Fitopatologia, Universidade Federal de Lavras, Lavras 37200-900, Brazil; mlucio@ufla.br; 3 Department of Plant Pathology, Kansas State University, Manhattan, KS 66506-0110, USA

**Keywords:** molecular identification, leaf sheath assay, sugarcane, red rot, Everglades Agriculture Area, Florida, *Colletotrichum falcatum* disease cycle

## Abstract

Red rot disease reduces sugarcane yield and impacts the sugar quality, posing an important threat to the sugarcane industry in Florida. Although *Colletotrichum falcatum*, the causal agent of red rot in Florida, was first reported in 1984 based on morphology, molecular and pathological data have remained limited, highlighting the critical need for comprehensive characterization. Thirteen isolates were obtained from three local sugarcane varieties in Belle Glade, Florida. Phylogenetic analyses of five genetic markers (ITS, *ACT*, *TUB2*, *GAPDH*, and *CHS-1*) confirmed all the strains as *C. falcatum*. In addition, the study documented the disease progression at the cellular level and assessed the pathogenicity of representative strains using the leaf sheath and whole-seedling inoculation methods. The varieties CP96-1252 and CP89-2143 showed greater host resistance. These findings represent the first report of *C. falcatum* causing red rot in southern Florida, offer valuable insights for/into red rot management, and provide a basis for future breeding programs to enhance sugarcane resistance to red rot disease.

## 1. Introduction

Sugarcane (*Saccharum* ssp.) is a global cash tropical grass crop cultivated across 26.3 million hectares, with a total production of approximately 1.9 billion tons [1]. In the 2022 and 2023 seasons, the United States (U.S.) ranked as the sixth largest sugarcane producer in the world [2]. Florida alone is responsible for more than 50% of the value of the sugar derived from sugarcane nationwide, generating approximately 712 million USD in 2020 [3]. Plant diseases significantly impact sugarcane production, especially in tropical and subtropical regions where warm temperatures and high humidity are conducive to disease development. Fungal pathogens can directly infect the interior of the sugarcane stalks, affecting yield, juice quality, and sugar content [4,5,6]. Among these, red rot stands out as one of the most important fungal diseases affecting sugarcane worldwide [7,8,9].

The foliar phase of this disease is characterized by reddish lesions that extend along the midrib and leaf blade, having a pale color at the center where the pathogen sporulates, producing conidia within acervuli [10,11]. The pathogen subsequently infects the cane stalks, which is known as the stalk phase [10], moving downwards through the vascular tissues, resulting in a reddish discoloration that can be observed by splitting the stalks longitudinally [12]. This infection leads to the repression of sucrose transporters [13] and the inversion of sucrose, compromising juice quality and reducing cane weight by 29 to 83% [14,15].

Three species of *Colletotrichum* (*C. falcatum*, *C. siamense*, and *C. plurivorum*) have been reported to be associated with sugarcane red rot in Brazil [9,16], although *Colletotrichum falcatum* is the predominant etiological agent in regions such as Bangladesh, Brazil, India, and the U.S. [8,9,16,17,18,19]. Despite its global importance, this disease remains poorly characterized, with critical knowledge gaps regarding the causative agent in the U.S. and its development in sugarcane living tissue.

Red rot was first observed in the U.S. in 1909 in Louisiana [20]. Since then, it has caused significant losses in sugarcane production in the southern U.S. [21]. In Florida, the first report of *C. falcatum* on sugarcane was produced based on morphological characteristics in 1984 [17]. Accurate pathogen identification is essential for developing effective management strategies [22]. However, morphological and physiological characteristics alone are often insufficient to differentiate between *Colletotrichum* species due to significant overlap [23]. For example, in 2009, six new species of *Colletotrichum* were identified causing disease on grasses, yet they showed no significant morphological similarities to other grass-associated *Colletotrichum* species, highlighting the necessity for molecular techniques in an accurate identification [24].

*Colletotrichum falcatum* has exhibited high variability in virulence, leading to the emergence of new pathotypes [5,8,25,26]. These new pathotypes pose a challenge to red rot management and breeding efforts, as they can overcome the resistance of existing varieties [25,26,27]. In Florida, the limited information on the resistance levels of local sugarcane varieties to red rot further complicates disease control.

Although extensive research has been conducted on the symptomatology and overall life cycle of *C. falcatum* in sugarcane, the majority of studies have focused on the stalk phase [22,28,29,30]. Consequently, our knowledge of the pathogen development within sugarcane leaves remains limited. A comprehensive understanding of the foliar phase is essential for elucidating the host–pathogen interaction during leaf infection and ultimately developing more effective management strategies against red rot [31,32].

Given the limited understanding of *Colletotrichum* species and the gaps in knowledge regarding their pathogenicity in Florida, there is a critical need to perform the molecular characterization and assess the pathogenicity of the *Colletotrichum* species associated with sugarcane in southern Florida. Therefore, the aims of this study were to (i) conduct a multilocus molecular phylogenetic analysis of *Colletotrichum* strains from southern Florida, (ii) investigate the foliar phase of *C. falcatum* at the cellular level, and (iii) assess the pathogenicity of representative isolates on three commercial sugarcane varieties grown in the region. These findings are vital for improving red rot management strategies and enhancing sugarcane resistance to this devastating disease.

## 2. Materials and Methods

### 2.1. Fungal Isolation

Sugarcane leaf samples were collected in 2022 and 2023 from plants in field from varieties CL85-1040, CP96-1252, and CP89-2143 exhibiting symptoms of red rot at the University of Florida Everglades Research and Education Center (EREC), Belle Glade, Florida (26°40′03″ N 80°38′06″ W). One symptomatic leaf was collected per plant, resulting in a total of 13 leaves for fungal isolation. Triangular fragments of healthy and symptomatic tissue were surface sterilized in 70% ethanol for 30 s and in 1.2% of sodium hypochlorite for 3 min, washed three times in sterile distilled water (SDW), and dried in a sterile paper towel. Four fragments of each leaf were plated in potato dextrose agar (PDA) (BD Difco™, New York, NY, USA) amended with 1% (*v*/*v*) neomycin-penicillin-streptomycin (Sigma-Aldrich, St Louis, MO, USA) at 23 °C for 7 to 10 days [33].

Single spores were transferred to new PDA plates to establish pure cultures and stored in 50% glycerol (*v*/*v*) at −80 °C. A negative control was obtained from anthracnose lesions from the crown of celery, subjected to the same isolation process, and included in the analyses.

### 2.2. DNA Extraction, PCR, and Sequencing

Genomic DNA of the 14 strains collected from the three sugarcane varieties CL85-1040 (*n* = 3), CP96-1252 (*n* = 6), and CP89-2143 (*n* = 4), and from the celery (*n* = 1), was extracted using 50 mg of fungal mycelia grown on PDA for 2 weeks at 23 °C under continuous fluorescent light. The DNA was extracted using the SYNERGY™ 2.0 DNA Extraction Kit (OPS Diagnostics, Lebanon, NJ, USA), following the manufacturer’s instructions. The total DNA obtained was eluted in 50 µL of Monarch^®^ DNA Elution Buffer (New England Biolabs, Ipswich, MA, USA). The final DNA concentration was measured using a NanoDropTM2000/2000c Spectrophotometer (Thermo Fisher Scientific, Waltham, MA, USA). Primers used in the study were synthesized by Integrated DNA Technologies (IDT^®^, Coralville, IA, USA) (Table 1). Polymerase chain reaction (PCR) was performed in a 25 µL reaction volume containing 1 µL of DNA (25 ng/µL), 12.5 µL o1f GoTaq^®^ Green Master Mix Kit (PROMEGA, Madison, WI, USA), and 11.5 µL of water. The cycling conditions were initial denaturation at 95 °C for 4 min, followed by 35 cycles of denaturation at 95 °C for 45 s; annealing at 51 °C, 59 °C, 56 °C, 58 °C, and 59 °C for ITS, *ACT*, *TUB2*, *GAPDH*, and *CHS-1*, respectively, for 45 s; extension at 72 °C for 59 s; and a final extension at 72 °C for 10 min. Following PCR amplification, the resulting DNA products were visualized on a 1% agarose gel stained with SYBR Safe (Thermo Fisher Scientific, Waltham, MA, USA). The amplified DNA was purified before Sanger sequencing on the ABI 3730XL sequencer at Molecular Cloning Laboratories (MCLAB, South San Franciso, CA, USA).

### 2.3. Phylogenetic Analyses

In addition to the 14 strains obtained in this study, 18 reference sequences were retrieved from NCBI GenBank, totaling 32 strains used in the phylogenetic analyses (Table 2). Raw DNA sequences from the 14 strains were manually annotated for each of the five gene regions (ITS, *ACT*, *TUB2*, *GAPDH*, and *CHS-1*) to ensure the accuracy and high quality of the final consensus sequences before performing a BLAST search and aligning them using Geneious Prime 6.0.6. The sequence alignment was performed with MAFFT v. 7.407_1, and the most conserved regions were filtered by Gblocks v. 0.91.1 integrated within NGPhylogeny.fr [39,40,41]. Phylogenetic trees were constructed using both maximum likelihood (ML) estimated with MEGA v.11 [42] and the Bayesian inference (BI) performed with MrBayes v. 3.2.6. For the ML analysis, the best-fit models K2 + G + I (ITS), K2 + I (*ACT*), K2 + G (*TUB2*, *GAPDH* and *CHS-1*), and TN93 + G (concatenated tree) were selected based on the Bayesian information criterion (BIC), with 1000 bootstrap replicates [43]. The BI analysis employed SYM + I + G (ITS), HKY + I (*ACT*), HKY + G (*TUB2*, *GAPDH*), and GTR + G (*CHS-1*) substitution models based on the Akaike information criterion (AIC) and utilized a Markov Chain Monte Carlo (MCMC) search with 5,000,000 generations, sampling trees every 100 generations [44]. The phylogenetic trees of the individual and concatenated genes were developed using iTOL (https://itol.embl.de/, accessed on 23 October 2024). *Colletotrichum boninense* was used as the outgroup. All the sequences resulting from this study of *Colletotrichum* spp. from sugarcane (*n* = 13) and one from celery (*n* = 1) were deposited in GenBank (https://www.ncbi.nlm.nih.gov/genbank/, accessed on 23 October 2024) under the following accession numbers: PP782092 to PP782105 (ITS), PP855401 to PP855414 (*ACT*), PQ349366 to PQ349379 (*TUB2*), PQ349380 to PQ349393 (*GAPDH*), and PQ349352 to PQ349365 (*CHS-1*) (Table 2).

### 2.4. Fungal Inoculum

*Colletotrichum* strains were grown on PDA at 23 °C under continuous fluorescent light for 2 weeks. The spores were scraped using a sterilized mini pestle in SDW and filtered in a double layer of cotton gauze [56,57]. The spore suspension was centrifuged three times at 3500× *g*, 25 °C for 5 min, and the SDW was changed between the centrifugation cycles [56,57]. A spore concentration was adjusted to 10^5^ spores/mL and immediately used for both leaf sheath assays and seedling spray inoculations [56,57].

### 2.5. Sugarcane Plants

Single-eye pieces were obtained from sugarcane stalks (CL85-1040, CP96-1252, and CP89-2143) and planted 2 cm apart in a rectangular tray (40 × 50 cm) filled with a potting mixture (Professional Growing mix, Sun Gro Horticulture, Agawam, MA, USA). One-week-old seedlings were transferred to pots (21 × 22 cm) filled with a potting mixture for the whole-seedling spray inoculation experiment. The variety CL85-1040 used in the leaf sheath assay was kept in the trays, and 1-month-old seedlings were cut at the soil line and brought to the laboratory for the leaf sheath assay. Osmocote fertilizer (14-14-14) (ICL growing solutions, Tel Aviv, Israel) was added 1 week after planting the seed eyes and seedlings. The plants were kept in the greenhouse at ambient temperature, under 1 min irrigation twice a day, and then they were used to set up the whole plant or the leaf sheath experiments.

### 2.6. Leaf Sheath Colonization Assay

The colonization of *C. falcatum* on the cellular level was investigated using the most susceptible variety, CL85-1040, and the most virulent strain, KX144, in a time-course experiment. Detached sugarcane leaf sheaths were collected and prepared according to Belisário et al. [57] with modifications. Briefly, the leaf sheaths were inoculated with 100 μL of KX144 spore suspension (10^5^ spores/mL) and incubated at 21 ± 1 °C under continuous fluorescent light in 100% humidity. For negative controls, we used two treatments: (1) inoculation with the non-pathogenic *Colletotrichum theobromicola* isolate KX164, originally isolated from celery, and (2) inoculation with SDW. Treatments were arranged in a completely randomized design with four replicates. The evaluation was performed at 12, 24, 36, 48, 60, 72, and 146 h after inoculation (hai). A total of 50 infection sites per repetition were evaluated randomly using the Leica ICC50 E microscope (Leica Microsystems, Leica, Wetzlar, Germany) and visualized using Leica Application Suite LAS EZ v. 3.4.0 software, following the diagrammatic scale developed by Xavier et al. [56].

### 2.7. Pathogenicity Tests

#### 2.7.1. Leaf Sheath Pathogenicity Assay

One fungal strain from each variety (KX144, KX145, and KX146 from CL85-1040, CP96-1252, and CP89-2143, respectively) was selected for the leaf sheath pathogenicity assay to inoculate on the ‘CL85-1040’. Additionally, KX164 (negative control) was included. All the plants were inoculated with a spore suspension of 10^5^ spores/mL. Inoculation with SDW was used as a control. The evaluation was performed at 60 hai based on 50 infection sites per repetition under the light microscope based on the scale developed by Xavier et al. [56] with modifications. Briefly, it was performed in two different ways: (i) attending to how the pathogen was colonizing the plant cells, and (ii) attending to how the plant was responding against the pathogen by restricting its growth to one cell or by the presence of red vesicles at the infection sites.

#### 2.7.2. Whole-Seedling Spray Inoculation

The pathogenicity of the strains (KX144, KX145, and KX146) from the local sugarcane varieties (CL85-1040, CP96-1252, and CP89-2143, respectively) was assessed by spray-inoculating each variety with a spore suspension of 10^5^ spores/mL in 1% (*v*/*v*) Tween 20. The strain KX164 from celery and Tween 20 were used as the negative control. After spray inoculation, the plants were bagged with a clear bag and maintained in a 100% humidity chamber for 48 h. After that, the bags were removed, and the plants were kept on the greenhouse benches, where they were irrigated twice a day at 7 am and 7 pm for 1 min each time. Treatments were arranged in a completely randomized block design with four replicates and two plants per pot per replicate. Disease severity was evaluated weekly after disease symptoms were observed, and re-isolation was performed after the last assessment to fulfill Koch’s postulate. The experiment was performed three times (October and November 2023 and February 2024).

#### 2.7.3. Statistical Analysis

Statistical analyses were performed in R version 4.3.2 [58]. The two repetitions of the leaf sheath virulence experiment were analyzed through a cumulative logistic mixed model (CLMM) using the *clmm* function of the ‘ordinal’ package v. 4.1 [59]. This approach appropriately models ordinal data [60,61]. Experiments, fungi, and their interaction were treated as fixed effects, and replicates within experiments were treated as random effects. In both pathogenicity tests’ statistical analyses ((whole-seedling spray inoculation and leaf sheath pathogenicity assay), the means and 95% confidence intervals of each group were estimated using the *emmeans* function of the ‘emmeans’ package v. 1.10.4 [62]. Statistically significant pairwise differences (*p* < 0.05 following Tukey’s adjustment) were visualized using a compact letter display via the *cld* function of the ‘multcomp’ package v. 1.4.26 [63]. All graphs were created using functions from the ‘tidyverse’ package v. 2.0.0 [64] and ‘ggplot2’ package v. 3.5.1 [65].

The AUDPC values resultant from the three repetitions of the whole-seedling spray inoculation experiment were analyzed through generalized linear mixed models (GLMMs) using the function *glmmTMB* of the ‘glmmTMB’ package v. 1.1.9 [66]. A Tweedie distribution with a log link function was employed to account for zero-inflated data due to negative controls. Variety, fungal strain, and their interaction were included as fixed effects, while experiment and experimental unit were included as random effects. Model fit was assessed via plotting the simulated quantile residuals using the *simulateResiduals* function from the ‘DHARMa’ package v. 0.4.6 [67]. Those figures and comparisons by Akaike’s information criteria (AIC) subsequently confirmed that model fit was improved by modeling dispersion as varying among the different strains. A Wald χ^2^ test was then performed, followed by the calculation of estimated marginal means, 95% confidence intervals, and pairwise comparisons (using Tukey’s adjustment) among fungal strains within each variety, and for each variety averaged across all strains.

## 3. Results

### 3.1. Colletotrichum *spp.* in Southern Florida

Red rot was observed on local sugarcane varieties during the summers of 2022 and 2023, at the Everglades Research and Education Center, University of Florida, in Belle Glade, Florida. The average disease incidence in the field was approximately 60–80%on varieties not treated with pesticides. Symptoms of red rot included reddish lesions on the midrib, with a pale center in which the pathogen sporulated, producing black dots corresponding to acervuli when visualized at higher magnification. Thirteen isolates collected from three sugarcane varieties [CL85-1040 (*n* = 3), CP96-1252 (*n* = 6), and CP89-2143 (*n* = 4)] were identified as *Colletotrichum* spp. based on morphological traits such as the presence of acervuli with setae, banana-shaped spores, and gray mycelium with orange sporulation on PDA media.

### 3.2. Molecular Characterization

**Figure 1:**
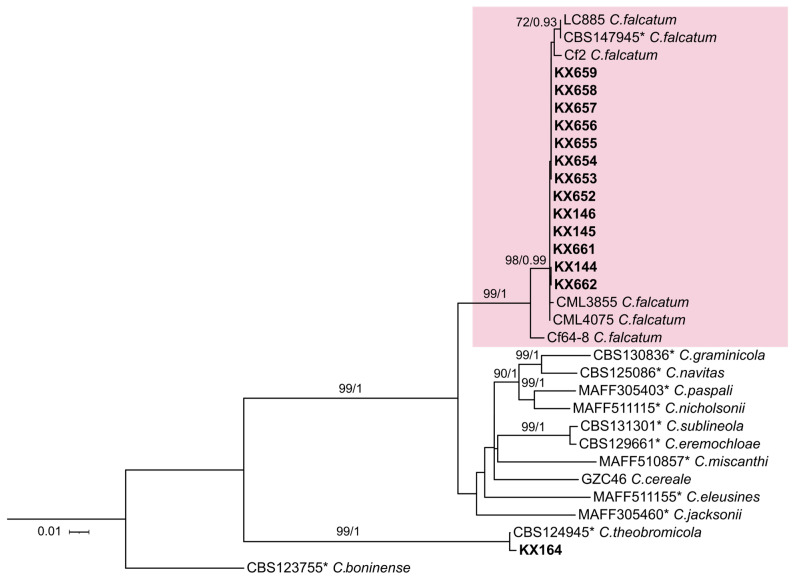
The consensus phylogenetic tree of 32 *Colletotrichum* species strains was generated based on maximum likelihood (ML) and Bayesian inference (BI) analyses of combined ITS, *ACT*, *TUB2*, *GAPDH*, and *CHS-1* gene sequences. Bootstrap support values and Bayesian posterior probabilities (only values ≥ 70% and ≥ 0.9, respectively) are shown at the nodes. Isolates from this study are highlighted in bold, and ex-type cultures are marked with an asterisk. *Colletotrichum boninense* was used as the outgroup.

The final alignment of the concatenated gene sequences contained a total of 1568 bp (Figure 1) (ITS: 412 bp; *ACT*: 277 bp, *TUB2*: 497 bp, *GAPDH*: 121 bp, *CHS-1*: 261 bp), which were utilized in the phylogenetic analyses, considering alignment gaps and missing data from incomplete genes, representing 507 phylogenetically informative site patterns (ITS: 76, *ACT*: 121, *TUB2*: 200, *GAPDH*: 56, *CHS-1*: 54) (Figures S1–S5). Based on the concatenated alignment, our sugarcane strains from Florida had 99.8% similarity with the *C. falcatum* isolate from sugarcane CML4075 in Brazil [16]. Furthermore, the concatenated phylogenetic tree revealed that all 13 sugarcane strains from Florida clustered within the *Colletotrichum falcatum* clade alongside reference isolates of this species, confirming their species identification. All 13 strains had identical ITS, *ACT*, *TUB2*, *GAPDH*, and *CHS-1* sequences, except for KX145, KX146, KX652, and KX661, which exhibited a single nucleotide polymorphism at the same position in the *GAPDH* gene.

### 3.3. Leaf Sheath Development Assay

**Figure 2:**
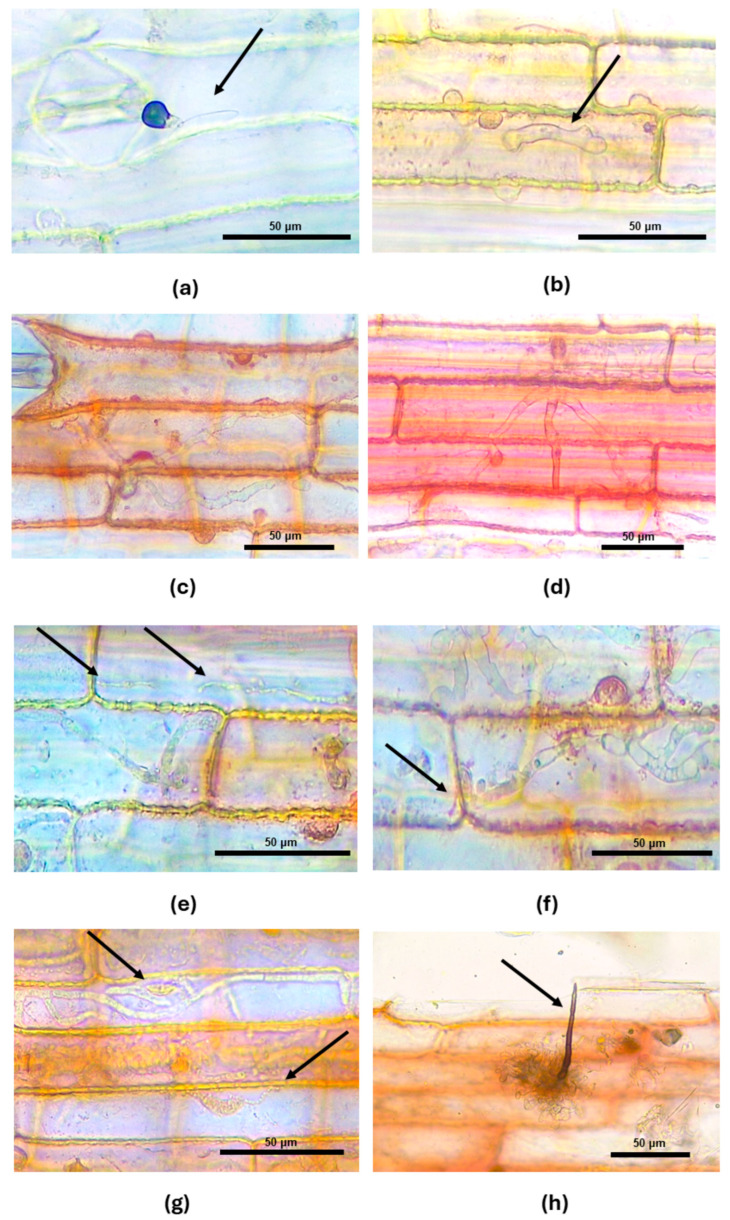
Time course of *C. falcatum* KX144 development in sugarcane leaf sheaths (CL85-1040). (**a**) Germinated spores with appressorium (black arrow) at 12 hai. (**b**) Primary hyphae (black arrow) in one cell at 12 hai. (**c**) Primary hyphae in two cells at 24 hai. (**d**) Primary hyphae in three or more cells at 36 hai. (**e**–**g**) Secondary hyphae (black arrows) and shifting from biotrophic to necrotrophic phase at 36-48 hai. (**h**) Acervulus with setae (black arrow) at 146 hai.

**Figure 3:**
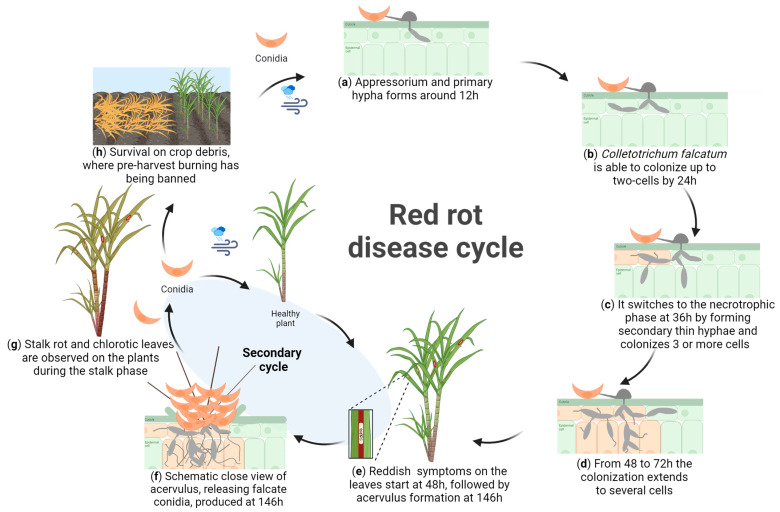
Scheme of red rot disease cycle on sugarcane: (**a**–**f**) Foliar phase: (**a**) *Colletotrichum falcatum* spore germinates, forms appressorium, and primary hyphae by 12 hai, (**b**) primary hyphae colonizes up to two cells by 24 hai, (**c**) thin secondary hyphae forms by 36 hai, (**d**) colonization of thick and thin hyphae continues to several cells from 48 to 72 hai, (**e**) first reddish lesions visible on the leaves at 48 hai, acervuli are produced around 146 hai. (**e**–**h**) The stalk phase [28,68]: (**e**) *Colletotrichum falcatum* spores infect the stalk, initiating the colonization of internal tissues. (**f**) The symptoms of red rot in the stalks become visible, and (**g**) may lead to stalk collapse. During the secondary cycle, spores can be released by rain splashes and disseminated by wind to healthy plants in the same season or (**h**) survive in crop debris [68], becoming a source of inoculum in subsequent seasons [28]. Created with BioRender.com.

At the earliest time point (12 hai), pathogen spores germinated, formed appressoria (Figure 2a and Figure 3a), and infected the host cells. In some instances, primary thick hyphae were already forming (Figure 2b and Figure 3a), indicating the start of biotrophic infection and colonization. By 24 hai, the pathogen had extended its colonization to two cells (Figure 2c and Figure 3b), and at 36 hai, it had spread to three or more cells (Figure 2d and Figure 3c). The formation of the thin secondary hyphae developing from primary thick hyphae was observed at 36 hai (Figure 2e,f and Figure 3c), marking the transition from biotrophic to necrotrophic infection. From 48 to 72 hai, extensive colonization by both thick and thin hyphae was evident across multiple host cells (Figure 2g and Figure 3d). Remarkably, by 146 hai (or 6 days after inoculation), the pathogen had completed its life cycle, with the formation of acervuli, from which conidia were formed and released (Figure 2h and Figure 3e,f).

### 3.4. Pathogenicity Tests

#### 3.4.1. Leaf Sheath Pathogenicity Assay

**Figure 4:**
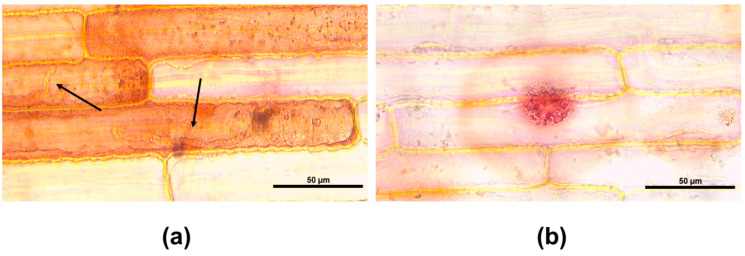
Plant responses to *Colletotrichum* infection in detached sugarcane leaf sheaths (CL85-1040) inoculated with strain KX164 from celery. (**a**) Primary hyphae (black arrows) in one cell. (**b**) Red vesicles around the infection site.

The leaf sheath time-course assay described above revealed that colonization beyond two host cells would lead to compatible interaction. Furthermore, no significant differences were observed between 48, 60, and 72 hai. Therefore, the virulence of strains KX144, KX145, and KX146 on the variety CL85-1040 was assessed at 60 hai. In addition, the assessment of plant responses revealed distinct infection sites characterized by hyphae constricted within a single cell (Figure 4a). Additionally, some sites were observed to be surrounded by red vesicles, indicating active defense mechanisms at play (Figure 4b).

**Figure 5:**
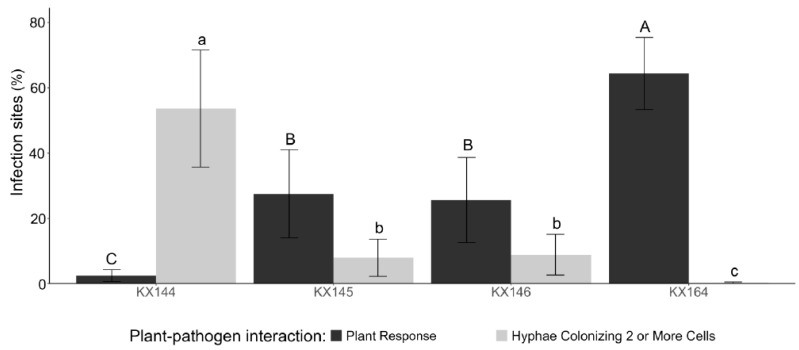
Plant response and pathogen colonization in sugarcane leaf sheaths (CL85-1040) at 60 hai. Different letters indicate significant differences (*p* < 0.05) based on the CLMM: uppercase letters for plant responses and lowercase letters for pathogen colonization.

There were no significant differences between the two experimental replications (*p* = 0.7247), allowing the data to be combined for further analysis. Our results indicated that strain KX144 exhibited the greatest virulence, with a higher capacity to colonize more than two cells (*p* < 0.0001) (Figure 5). Correspondingly, this strain triggered the lowest observed plant response. Conversely, the negative control (KX164) caused the strongest plant response. Strains KX145 and KX146 demonstrated similar behavior and were not statistically different from each other, suggesting a lower level of virulence compared to KX144 (Figure 5).

#### 3.4.2. Whole-Seedling Spray Inoculation

**Figure 6:**
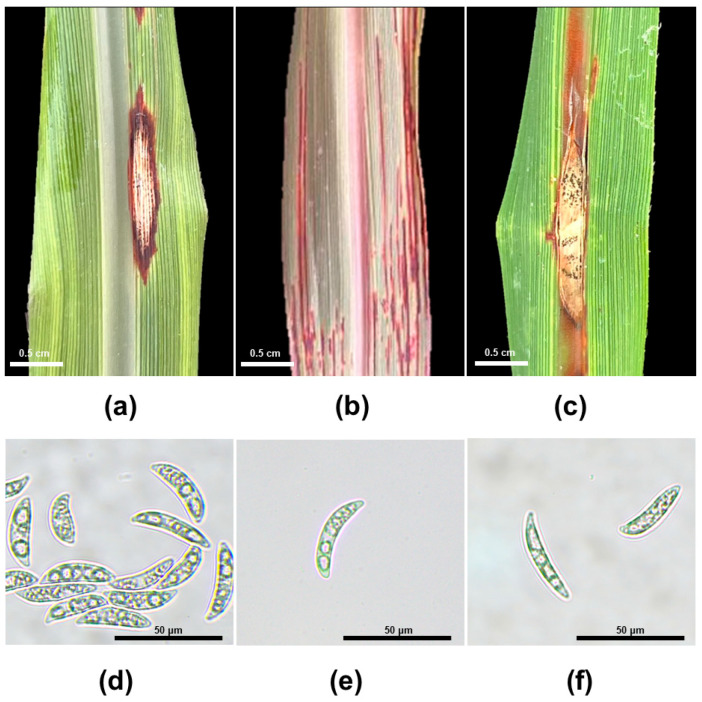
Symptoms of red rot in sugarcane varieties inoculated with their respective *Colletotrichum falcatum* strains: (**a**) KX144 on CL85-1040, (**b**) KX145 on CL96-1252, (**c**) KX146 on CL89-2143. Re-isolated banana-shaped conidia of each strain: (**d**) KX144, (**e**) KX145, (**f**) KX146.

The pathogenicity of Florida *Colletotrichum falcatum* strains was evaluated in sugarcane whole seedlings in the greenhouse. The first symptoms of red rot, characterized as small reddish lesions, were observed on the midrib and leaf blade at 48 hai, when the plants were removed from the humidity chambers. Overall, the symptoms gradually increase in size, forming pale necrotic lesions in the center, where black dots (acervuli) first appeared at 4, 5, and 7 days in experiments 1, 2, and 3, respectively, indicating that the pathogen can complete its life cycle as early as 4 days after inoculation (Figure 6). However, there were some specific symptoms that were characteristic among the isolates regardless of the sugarcane variety inoculated. Isolate KX144 caused oval-shaped lesions with a light pale center surrounded by dark red to brown borders on lesions found both on the leaf blade and midrib (Figure 6a). Plants inoculated with isolate KX146 displayed round lesions, red to orange, with a pale center primarily on the midrib (Figure 6c), whereas isolate KX145 induced the formation of fine, elongated reddish lesions mainly on the leaf blade of newly developed leaves (Figure 6b).

In summary, all three strains, KX144 from CL85-1040, KX145 from CP96-1252, and KX146 from CP89-2143, exhibited pathogenicity on all the sugarcane varieties tested in this study. Re-isolations from symptomatic leaves confirmed that red rot was caused by the inoculated strains. No pathogen was recovered from the control treatments. The presence of banana-shaped conidia (Figure 6d–f) in the re-isolated cultures fulfilled Koch’s postulate.

**Figure 7:**
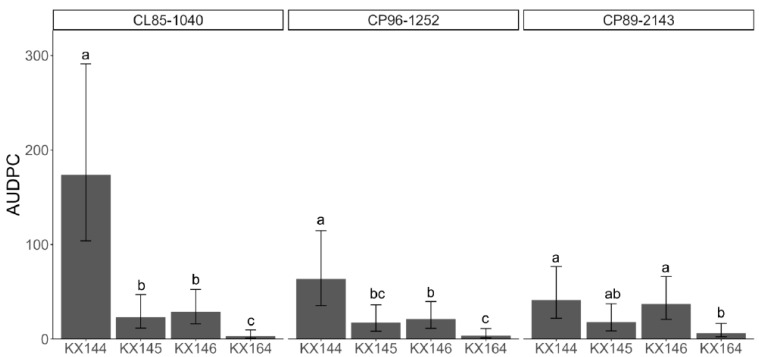
Area Under Disease Progress Curve (AUDPC) of three combined experiments. Different letters represent statistically different (*p* < 0.05) values calculated by the generalized linear mixed models (GLMMs).

The GLMM indicated that the Area Under Disease Progress Curve (AUDPC) was significantly affected by fungi (*p* < 0.0001) and fungi–variety interaction (*p* = 0.0014). Based on the AUDPC, strain KX144 isolated from variety CL85-1040 was the most virulent among the fungi tested. The exception was on the variety CP89-2143, in which there was no statistical difference among the evaluated strains. The strains KX145 and KX146 showed similar virulence among the varieties. The negative control KX164 was not significantly different from plants sprayed with water (Tween 1%).(Figure 7).

**Figure 8:**
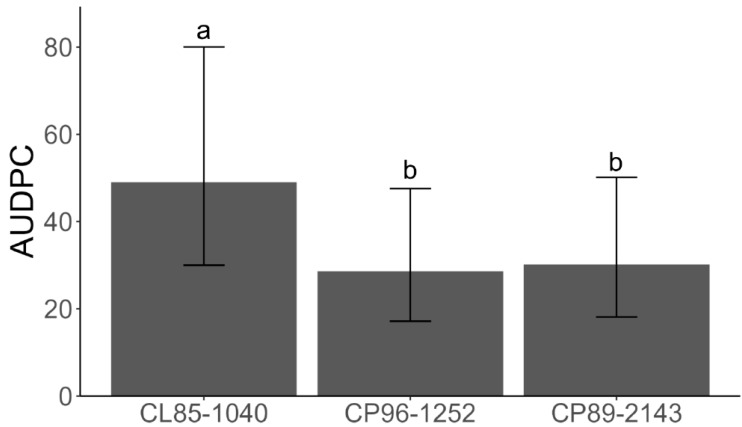
Area Under Disease Progress Curve (AUDPC) comparing sugarcane varieties, based on combined ratings for three *Colletotrichum falcatum* strains (KX144, KX145, and KX146). Different letters represent statistically different (*p* < 0.05) values determined by the generalized linear mixed models (GLMMs).

To assess resistance levels among the three sugarcane varieties, the AUDPC values were calculated for each variety based on the combined disease severity caused by all three strains (KX144, KX145, and KX146). Varieties CP96-1252 and CP89-2143 exhibited significantly lower AUDPC values compared to variety CL85-1040, indicating greater resistance to red rot (Figure 8).

## 4. Discussion

This study presents the first comprehensive characterization of *Colletotrichum falcatum* as the causative agent of red rot on sugarcane in southern Florida. Recent observations at the Everglades Research and Education Center in Belle Glade, Florida, have revealed a concerning incidence of red rot, with infection rates ranging from 60% to 80% during the summer months across local sugarcane varieties. Similar red rot outbreaks have also been reported in other major sugarcane-producing regions, such as India [30], suggesting the global relevance of this disease. Although red rot has been present in Florida for several years, its economic impact on sugarcane yields has remained minimal, especially when compared to other tropical regions like Brazil, where the disease has caused yield losses of up to 24.2% [68]. A key factor that may play a role in mitigating the impact of red rot in Florida is the pre-harvest practice of burning fields, which likely reduces pathogen inoculum by eliminating crop debris between seasons.

This study collected 13 *Colletotrichum* isolates for molecular and pathological characterization, addressing the lack of detailed data on *Colletotrichum* in the region. Multilocus molecular phylogenetic analysis revealed that all 13 isolates from symptomatic sugarcane leaves clustered within the *Colletotrichum falcatum* clade. This identification was supported by using key genetic markers—ITS, *TUB2*, *ACT*, *GAPDH*, and *CHS-1*—based on the established methodologies from earlier studies in the *Colletotrichum graminicola* complex [69,70]). Notably, at least two of these markers have also been employed in recent studies of *C. falcatum* from Pakistan, Bangladesh, and Brazil [8,16,71]. These findings highlight the reliability of these genetic markers in accurately identifying, assessing the genetic diversity of, and determining the evolutionary relationships within *C. falcatum* populations across different regions.

While most previous studies have focused on the stalk phase of red rot [22,28,29], the present study is the first to target the foliar phase of the fungal life cycle. The leaf sheath assays provide a unique view into the cellular interaction between pathogens and hosts, as seen in the *Colletotrichum sublineola*–sweet sorghum [56] and *Colletotrichum graminicola*–maize [57] pathosystems. We monitored the *C. falcatum* life cycle within sugarcane living tissue at seven time points and developed the red rot disease cycle from spore germination to pathogen sporulation. Our observations on the strain KX144 align with the established hemibiotrophic nature seen in other *Colletotrichum* species [32]. Initially, this fungus colonizes as a biotroph, acquiring nutrients from living host cells before switching to a necrotrophic phase, where it feeds on dead tissues [32]. Spores of *C. falcatum* germinate and form primary hyphae at 12 hai (Figure 2b and Figure 3a). This primary hypha is similar to haustoria in biotrophic pathogens [72], likely facilitating nutrient acquisition and the secretion of effector molecules into the host cell cytoplasm, promoting infection and/or inducing a plant response during the initial biotrophic phase [72,73]. By 24 hai, we observed colonization of up to two plant cells (Figure 2c and Figure 3b). This finding aligns with Xavier et al. [56], who proposed this threshold to separate compatible (≥2 colonized cells) from incompatible (restricted to one cell) interactions in their study of *C. sublineola* on sorghum plants. The transition from biotrophy to necrotrophy for strain KX144 was observed at 36 hai, marked by the development of secondary hyphae (Figure 2e,f and Figure 3c). Notably, the complete *C. falcatum* life cycle, including acervuli formation (conidia-bearing structures), was achieved within 146 hai (6 days) (Figure 2h and Figure 3e,f). These findings from the leaf sheath assay mirrored those observed from whole-seedling inoculations, where the life cycle was completed within 4 to 7 days. *Colletotrichum falcatum* manifests a significant impact on the sugarcane stalks in what is known as the stalk phase. Previous research indicates that *C. falcatum* spores can infect the stalk through wounds or runoff from the midrib, initiating colonization of the internal tissues (Figure 3e) [28]. As symptoms of red rot become visible in the stalks (Figure 3f), the disease may ultimately lead to stalk collapse (Figure 3g). Spores produced on the leaves, midrib, and stalks can overwinter on crop debris, contributing to the disease cycle by allowing falcate spores to be released by rain splashes and disseminated by wind to healthy plants in the same season or by surviving in crop debris to become a source of inoculum in subsequent seasons (Figure 3h) [28,68]. Understanding the complete disease is crucial for developing a management strategy that addresses both foliar and stalk impacts on sugarcane.

We reconstructed the key events of the *C. falcatum* foliar life cycle in sugarcane by systematically analyzing 50 infection sites per repetition. It is crucial to note that multiple infection processes often occur simultaneously, sometimes within the same leaf sheath. In some instances, plant cells exhibited strong responses to pathogen infection, while in others, the pathogen colonized several plant cells, as demonstrated in the virulence assay. This study significantly advances our understanding of *C. falcatum*’s foliar life cycle and pathogenicity in sugarcane. By employing the leaf sheath assay, we were able to identify key stages of infection in real time, capturing details of both the biotrophic and necrotrophic phases. This approach offers advantages over previous studies using GFP-tagged *C. falcatum* to explore host–pathogen interactions [74] as our method uses intact leaf sheaths, which minimizes biases introduced by the artificial injuries required in GFP assays. Furthermore, our study allowed us to observe the natural behavior of *C. falcatum* and host cells, providing a clearer view of their interactions.

To better understand the responses of sugarcane plants against *Colletotrichum*, we used a *C. theobromicola* isolate (KX164) from celery, which is non-pathogenic to sugarcane. These responses included hyphae confinement or red vesicle formation (Figure 4), serving as a marker for virulence assessment at the cellular level. Microscopic observations from the leaf sheath assay and macroscopic symptom development in the greenhouse experiments consistently demonstrated that strain KX144 exhibited the highest virulence (Figure 5 and Figure 7). It successfully colonized multiple cells and caused the most severe symptoms (oval lesions with dark borders) on the susceptible sugarcane variety CL85-1040. Conversely, stains KX145 and KX146 showed intermediate virulence, reflected in both their colonization ability and the disease they induced (Figure 5). Notably, all three strains exhibited distinct disease symptom profiles when inoculated on their original hosts or alternative sugarcane varieties (Figure 6), highlighting potential strain-specific interactions and the influence of host genotype on disease development. This specificity in *C. falcatum* has been associated with the breakdown of varieties due to the emergence of new pathotypes [25,26,75]. It could be attributed to many factors, such as host origin, adaptation to the host, environmental conditions [25], and different production of hydrolytic enzymes [8,76]. Genetic diversity in the *C. falcatum* population manifests as distinct pathotypes [8,25], which can complicate disease management and breeding for resistance. While the limited scope of this study (sampling and variety testing) restricts definitive conclusions, the observed variation in virulence among the three *C. falcatum* strains suggests the potential presence of different races in Florida. Further investigation is required to definitively identify which resistant genes are involved that could potentially be different races in this pathogen population. Additionally, the varieties CP96-1252 and CP89-2143 demonstrated greater resistance compared to CL85-1040 (Figure 8). Given that red rot resistance is one of the criteria for varietal resistance for sugarcane commercial cultivation in other regions [77], future studies on the plant-host interaction mechanisms underlying this resistance could provide valuable insights for breeding if red rot becomes an epidemic in southern Florida in the future.

In conclusion, only one species of *Colletotrichum*, *C. falcatum*, was found to be associated with sugarcane in southern Florida. Therefore, it is essential to increase the survey area to represent the entire sugarcane growing area in the state, as well as other states. Additionally, it is important to investigate if there are different races within this pathogen population in Florida, as we observed a high degree of pathological diversity among the three evaluated strains when causing red rot under greenhouse conditions on local sugarcane varieties. This diversity can have significant implications for breeding programs. Furthermore, screening a wider range of sugarcane varieties for resistance is crucial to developing effective management strategies. Future research should also focus on fungicide resistance, increasing sample sizes for more comprehensive analysis, and exploring further resistance mechanisms in sugarcane varieties.

## Figures and Tables

**Table 1:** Sequences, annealing temperature, and references of primers used to amplify ITS region and *ACT*, *TUB2*, *GAPDH*, and *CHS-1* genes.

Locus ^1^	Primer	Sequence (5′-3′)	Ta (°C) ^2^	PS ^3^	References
ITS	ITS1F	CTTGGTCATTTAGAGGAAGTAA	49.7	429 bp	[34]
	ITS4	TCCTCCGCTTATTATATGC	52.1		[35]
*ACT*	ACT-512	ATGTGCAAGGCCGGTTTCGC	61.7	252 bp	[36]
	ACT-783R	TACGAGTCCTTCTGGCCCAT	57.9		[36]
*TUB2*	Bt1	AACATGCGTGAGATTGTAAGT	52.3	750 bp	[37]
	Bt2	ACCCTCAGTGTAGT GACCCTTGGC	62.5		[37]
*GAPDH*	GDF1	GCCGTCAACGACCCCTTCATTGA	61.7	150 bp	[38]
	GDR1	GGGTGGAGTCGTACTTGAGCATGT	60.6		[38]
*CHS-1*	CHS79F	TGGGGCAAGGATGCTTGGAAGAAG	61.7	300 bp	[36]
	CHS354R	TGGAAGAACCATCTGTGAGAGTTG	56.6		[36]

^1^ ITS, internal transcribed spacer; *ACT*, actin; *TUB2*, β-tubulin; *GAPDH*, glyceraldehyde-3-phosphate dehydrogenase; *CHS-1*, Chitin synthase. ^2^ Ta, annealing temperature. ^3^ PS, product size; bp, base pair.

**Table 2:** Strains used in the molecular phylogenetic analyses.

					GenBank Accession Numbers					
Strain ^1^	Species	Host/Substrate	Collection Date ^2^	Country	ITS	*ACT*	*TUB2*	*GAPDH*	*CHS-1*	References
CML 3855	*C. falcatum*	*Sarccharum officinarum* sp.	N/A	Brazil	MW471107	MW455488				[16]
CML 4075	*C. falcatum*	*Sarccharum officinarum* sp.	N/A	Brazil	MW471109	MW455490				[16]
Cf2	*C. falcatum*	*Sarccharum officinarum* sp.	August-2022	China	PP217423	OR542861	OR542897			[45]
LC885	*C. falcatum*	*Sarccharum officinarum* sp.	N/A	China	HM171677	HM171665	HM171680	HM171671		[46]
Cf64-8	*C. falcatum*	*Sarccharum officinarum* sp.	N/A	India	FJ002093	FJ008099		FJ002020		[8]
CBS 147945 *	*C. falcatum*	*Sarccharum officinarum* sp.	N/A	Indonesia	JQ005772	JQ005835	JQ005856		JQ005793	[46]
CBS 130836 *	*C. gaminicola*	*Zea mays*	N/A	USA	DQ003110	JQ005830	JQ005851	MW740221	JQ005788	[47,48,49]
CBS 125086 *	*C. navitas*	*Panicum virgatum*	N/A	USA	GQ919067	JQ005832	JQ005853		JQ005790	[48,50]
MAFF 511115 *	*C. nicholsonii*	*Paspalum dilatatum*	N/A	Japan	EU554126	JQ005833	JQ005854	MW740226	JQ005791	[48,49,51] https://www.tandfonline.com/doi/full/10.1080/00275514.2021.2008765 accessed on 9 August 2023
CBS 131301 *	*C. sublineola*	*Sorghum bicolor*	N/A	Burkina Faso	DQ003114	JQ005834	JQ005855	MW740232	JQ005792	[32,47,49]
MAFF 305403 *	*C. paspali*	*Paspalum notatum*	N/A	Japan	EU554100	JX519235	JX519244	MW740210	JX519227	[49,51,52]
CBS 129661 *	*C. eremochloae*	*Eremochloa ophiuroides*	N/A	USA	JQ478447	JX519236	JX519245		JX519228	[52]
MAFF 510857 *	*C. miscanthi*	*Miscanthus sinensis*	N/A	Japan	EU554121	JX519237	JX519246	MW740225	JX519229	[49,51,52]
MAFF 511155 *	*C. eleusines*	*Eleusine indica*	N/A	Japan	EU554131	JX519234	JX519243	MW740217	JX519226	[49,51,52]
GZC46	*C. cereale*	*Lolium multiflorum*	June-2022	China	OR976053	OR995786	OR995792	OR995768	OR995774	[53]
MAFF 305460 *	*C. jacksonii*	*Echinochloa esculenta*	N/A	Japan	EU554108	JX519233	JX519241	MW740224	JX519224	[49,51,52]
CBS 124945 *	*C. theobromicola*	*Theobroma cacao*	N/A	Panama	JX010294	JX009444	JX010447	JX010006	JX009869.1	[54]
CBS 123755 *	*C. boninense*	*Crinum asiaticum* var.	N/A	Japan	JQ005153	JQ005501	JQ005588	JQ005240	JQ005327	[55]
KX144	*C. falcatum*	*Sarccharum officinarum* sp./CL85-1040	August-2022	Florida, USA	PP782093	PP855401	PQ349366	PQ349380	PQ349352	First report in this study
KX652	*C. falcatum*	*Sarccharum officinarum* sp./CL85-1040	July-2023	Florida, USA	PP782096	PP855404	PQ349369	PQ349383	PQ349355	First report in this study
KX653	*C. falcatum*	*Sarccharum officinarum* sp./CL85-1040	July-2023	Florida, USA	PP782097	PP855405	PQ349370	PQ349384	PQ349356	First report in this study
KX145	*C. falcatum*	*Sarccharum officinarum* sp./CP96-1252	September-2022	Florida, USA	PP782094	PP855402	PQ349367	PQ349381	PQ349353	First report in this study
KX657	*C. falcatum*	*Sarccharum officinarum* sp./CP96-1252	July-2023	Florida, USA	PP782101	PP855408	PQ349374	PQ349388	PQ349360	First report in this study
KX658	*C. falcatum*	*Sarccharum officinarum* sp./CP96-1252	July-2023	Florida, USA	PP782102	PP855409	PQ349375	PQ349389	PQ349361	First report in this study
KX659	*C. falcatum*	*Sarccharum officinarum* sp./CP96-1252	July-2023	Florida, USA	PP782103	PP855410	PQ349376	PQ349390	PQ349362	First report in this study
KX661	*C. falcatum*	*Sarccharum officinarum* sp./CP96-1252	July-2023	Florida, USA	PP782104	PP855411	PQ349377	PQ349391	PQ349363	First report in this study
KX662	*C. falcatum*	*Sarccharum officinarum* sp./CP96-1252	July-2023	Florida, USA	PP782105	PP855412	PQ349378	PQ349392	PQ349364	First report in this study
KX146	*C. falcatum*	*Sarccharum officinarum* sp./CP89-2143	September-2022	Florida, USA	PP782095	PP855403	PQ349368	PQ349382	PQ349354	First report in this study
KX654	*C. falcatum*	*Sarccharum officinarum* sp./CP89-2143	July-2023	Florida, USA	PP782098	PP855406	PQ349371	PQ349385	PQ349357	First report in this study
KX655	*C. falcatum*	*Sarccharum officinarum* sp./CP89-2143	July-2023	Florida, USA	PP782099	PP855407	PQ349372	PQ349386	PQ349358	First report in this study
KX656	*C. falcatum*	*Sarccharum officinarum* sp./CP89-2143	July-2023	Florida, USA	PP782100	PP855413	PQ349373	PQ349387	PQ349359	First report in this study
KX164	*C. theobromicola*	*Apium graveolens*	December-2022	Florida, USA	PP782092	PP855414	PQ349379	PQ349393	PQ349365	First report in this study

^1^ *, ex-type cultures. ^2^ N/A, not available.

## Data Availability

The original contributions presented in the study are included in the article/Supplementary Materials; further inquiries can be directed to the corresponding author.

## Supplementary Materials

The following supporting information can be downloaded at https://www.mdpi.com/article/10.3390/jof10110742/s1. Figure S1: Maximum likelihood (ML) tree of 32 *Colletotrichum* spp. strains based on ITS region. The tree generated by Bayesian inference (BI) had a similar topology. Figure S2: Maximum likelihood (ML) tree of 32 *Colletotrichum* spp. strains based on *ACT* gene. The tree generated by Bayesian inference (BI) had a similar topology. Figure S3: Maximum likelihood (ML) tree of 29 *Colletotrichum* spp. strains based on *TUB2* gene. The tree generated by Bayesian inference (BI) had a similar topology. Figure S4: Maximum likelihood (ML) tree of 26 *Colletotrichum* spp. strains based on *GAPDH* gene. The tree generated by Bayesian inference (BI) had a similar topology. Figure S5: Maximum likelihood (ML) tree of 27 *Colletotrichum* spp. strains based on *CHS-1* gene. The tree generated by Bayesian inference (BI) had a similar topology.
