# Supplementary material for: Molecular Characterization and Pathogenicity of Colletotrichum falcatum Causing Red Rot on Sugarcane in Southern Florida
Source: J Fungi (Basel). 2024 Oct 27;10(11):742. doi: 10.3390/jof10110742 (PMC11595498; doi:10.3390/jof10110742)
Supplement: Supplementary file 1 [file jof-10-00742-s001.zip › jof-3150632-supplementary.pdf]

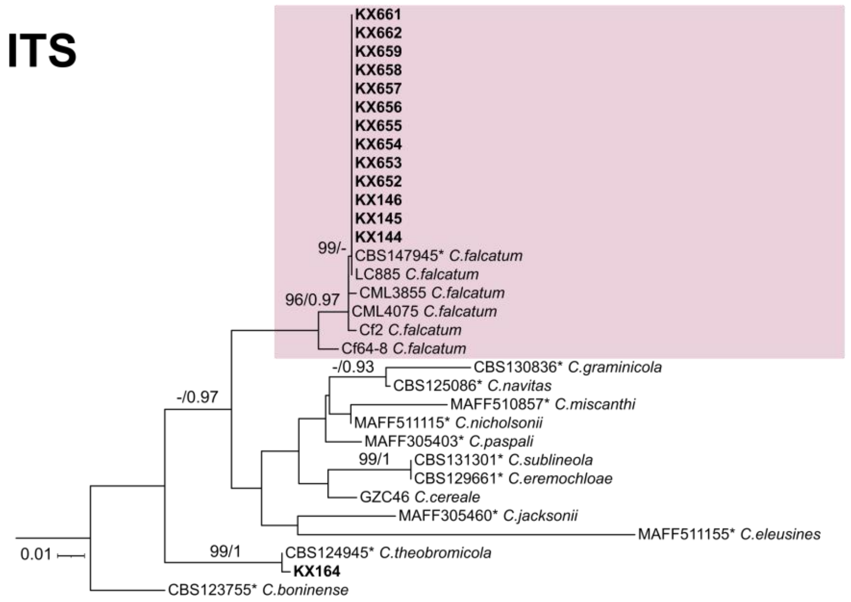

Figure S1. Maximum likelihood (ML) tree of 32 *Colletotrichum* spp. strains based on ITS region. The tree generated by Bayesian inference (BI) had a similar topology.

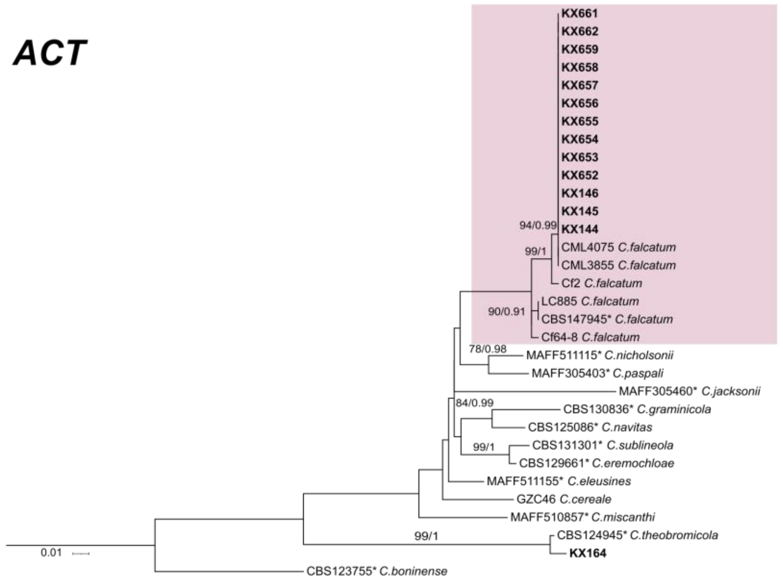

Figure S2. Maximum likelihood (ML) tree of 32 *Colletotrichum* spp. strains based on *ACT* gene. The tree generated by Bayesian inference (BI) had a similar topology.

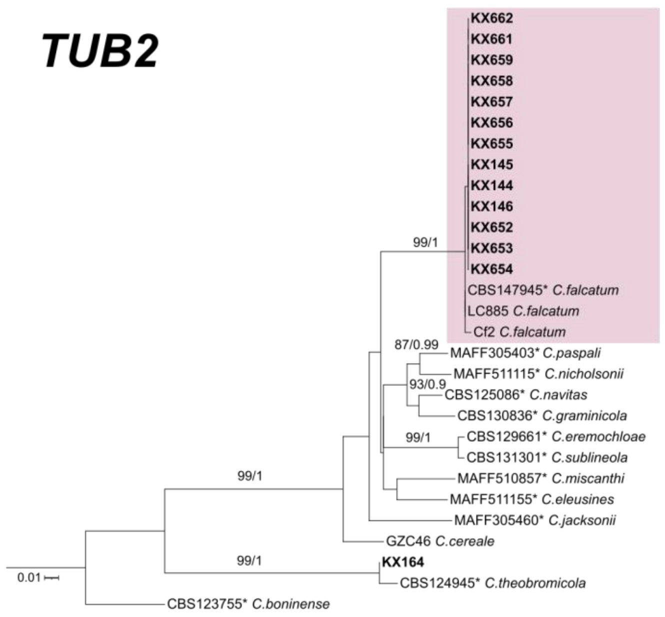

Figure S3. Maximum likelihood (ML) tree of 29 *Colletotrichum* spp. strains based on *TUB2* gene. The tree generated by Bayesian inference (BI) had a similar topology.

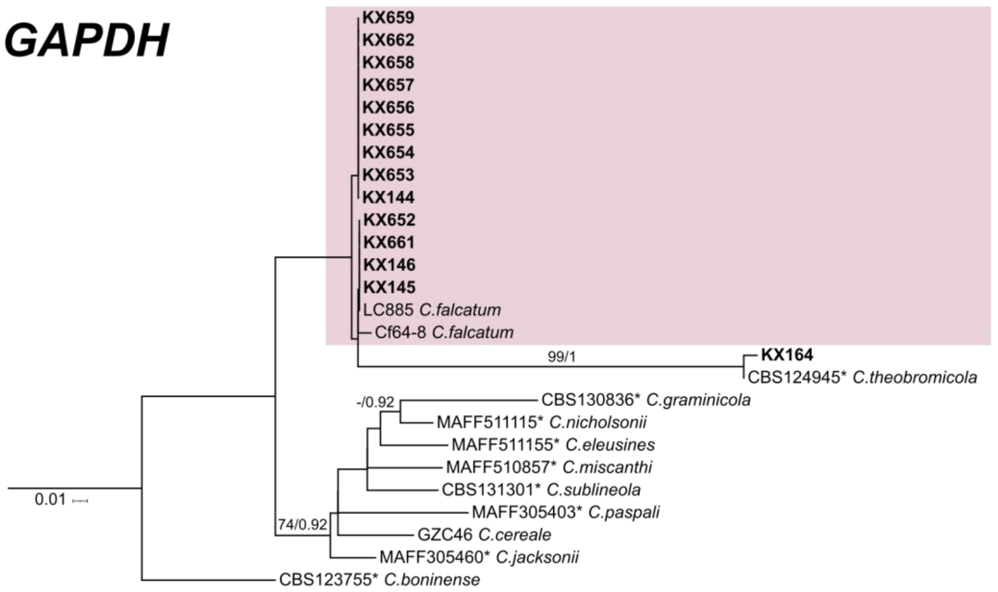

Figure S4. Maximum likelihood (ML) tree of 26 *Colletotrichum* spp. strains based on *GAPDH* gene. The tree generated by Bayesian inference (BI) had a similar topology.

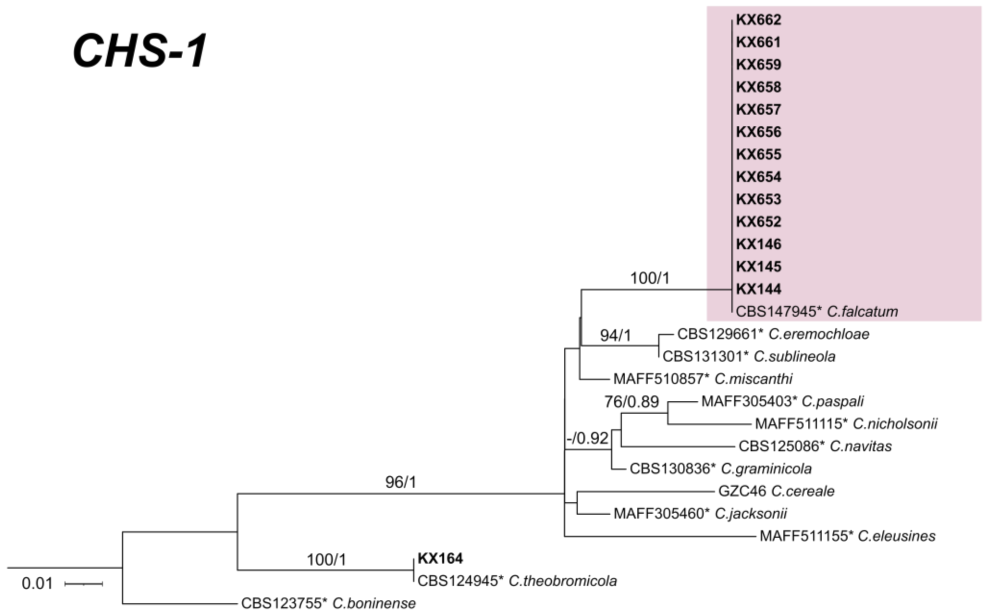

Figure S5. Maximum likelihood (ML) tree of 27 *Colletotrichum* spp. strains based on *CHS-1* gene. The tree generated by Bayesian inference (BI) had a similar topology.
